# *Euglena* sp. and its associated prokaryotic community degrade light crude oil

**DOI:** 10.1111/jpy.70227

**Published:** 2026-09-08

**Authors:** Michelle Kulbatzki, Hilal Kaya, Sabina Marks, Lisa Voskuhl

**Affiliations:** ^1^ Faculty of Chemistry, Environmental Microbiology and Biotechnology (EMB), Microbiology of Ecotones University of Duisburg‐Essen Essen Germany; ^2^ Faculty of Biology, ZMB, Chemische Biologie University of Duisburg‐Essen Essen Germany; ^3^ Centre for Water and Environmental Research (ZWU) University of Duisburg‐Essen Essen Germany

**Keywords:** algae, microalgae, molecular, symbiosis

## Abstract

Oil enters the environment through natural and anthropogenic sources and poses a serious threat to water and soil. We hypothesized that algae and their associated prokaryotic communities from natural oil seeps are robust systems capable of surviving and degrading anthropogenic oil spills. We isolated *Euglena* sp. together with its closely associated prokaryotes from a natural oil seep in Germany and tested its oil degradation potential in laboratory cultivation experiments. Oil mineralization was monitored using reverse stable isotope labeling (RSIL), and the associated microbiome was characterized by 16S rRNA gene amplicon sequencing over a 76‐day incubation. Dark incubations of *Euglena* sp. showed significantly higher oil degradation than day/night light cycle incubations. The RSIL data and microbial community dynamics indicated that oil degradation was primarily driven by *Euglena* sp. itself rather than by the associated prokaryotes. We conclude that the *Euglena* sp. culture SLN082 is a promising model for studying algal oil degradation and has potential for bioremediation applications. Comprehensive information on *Euglena* sp. isolation and experimental protocols, such as cultivation, RSIL measurements, DNA extraction and 16S rRNA gene amplicon sequencing, and subsequent bioinformatic analyses, is available in the supplementary text.

Oil spills into the environment occur all over the world, arising from natural and anthropogenic activities (Dong et al., 2022). Although anthropogenic oil spills have been extensively studied, natural oil seeps, particularly those occurring in terrestrial and freshwater environments, remain comparatively overlooked (Walter et al., 2026), even though they offer valuable insights into how natural ecosystems cope with hydrocarbon inputs. Such habitats are expected to host microbial and eukaryotic communities that have adapted to tolerate and degrade hydrocarbons due to their long‐term exposure. We here isolated *Euglena* sp. strain SLN082 from a soil sample next to a natural oil seep in Germany (Figures S1 and S2) with the aim of testing whether this microalga together with its associated prokaryotes are capable of surviving oil exposure and conducting oil biodegradation when exposed to an oil spill. *Euglena* sp. is a mixotrophic organism belonging to the phylum Euglenophyta, frequently occurring in nutrient‐rich freshwater habitats, brackish waters, soils, and wastewaters (Leander et al., 2017). *Euglena* can remove organic matter and heavy metals from wastewater (Rehman, 2011), and its extracellular metabolites can act as stimulants for diesel degradation by *Pseudomonas* (Chen et al., 2025). It was reported that individual and mixed hydrocarbons were harmful to growth by altering cell morphology and decreasing chlorophyll content (Kottuparambil & Park, 2019; Lukáčová et al., 2023; Peng et al., 2015). However, to the best of our knowledge, direct evidence on biodegradation of complex crude oil by *Euglena* and/or its associated microbiome does not exist.

Here, we report that non‐axenic *Euglena* sp. (SLN082) culture is capable of rapid crude oil biodegradation. Biodegradation experiments containing *Euglena* sp. (SLN082) and its associated microbiome were set up with and without the addition of light crude oil, hereafter referred to as light oil. Light oil is characterized by a relatively low density and high American Petroleum Institute (API) gravity (>31°; here: 37°), indicating a higher proportion of short‐chain hydrocarbons and lower viscosity compared to heavy crude oil. All conditions were incubated either under a day/night light cycle or in complete darkness to assess the contributions of phototrophic versus heterotrophic metabolism in *Euglena* (Data S1). The incubations were monitored over 76 days and were sampled nine times during that time (Data S2). Changes in pH were recorded throughout the experiment (Data S3). Oil mineralization to CO_2_ was quantified using the reverse stable isotope labeling (RSIL) method (Beilig et al., 2024, 2025; Dong et al., 2019; Pannekens et al., 2021), with raw data and calculations provided in Data S4, CO_2_ production plots in Data S5, and an exemplary RSIL calculation in Data S6 (see also Supplementary Text). Changes in the prokaryotic microbial community composition were analyzed by 16S rRNA gene amplicon sequencing, with metadata, taxonomy, amplicon sequence variant abundances, alpha diversity metrics, and mock community data provided in Data S7–S12.

After 3 days, visible changes in the oil phase were observed, characterized by the formation of oil flocs floating in the culture. Similar phenomena have been reported from marine oil‐contaminated environments as marine oil‐snow formation (Passow et al., 2012), which is typically associated with microbial extracellular polymeric substances (Sun et al., 2018). This aggregation reflects physical restructuring of the oil rather than direct evidence of chemical degradation. Oil snow formed most prominently under day/night light conditions, was reduced in dark incubations, and did not occur in abiotic controls (Figure 1a). Despite the higher emulsification in the day/night incubation, higher oil degradation rates were detectable in the dark incubations where no photosynthesis was possible and *Euglena* had to switch to a heterotrophic metabolism (Figure 1b,c).

**FIGURE 1:**
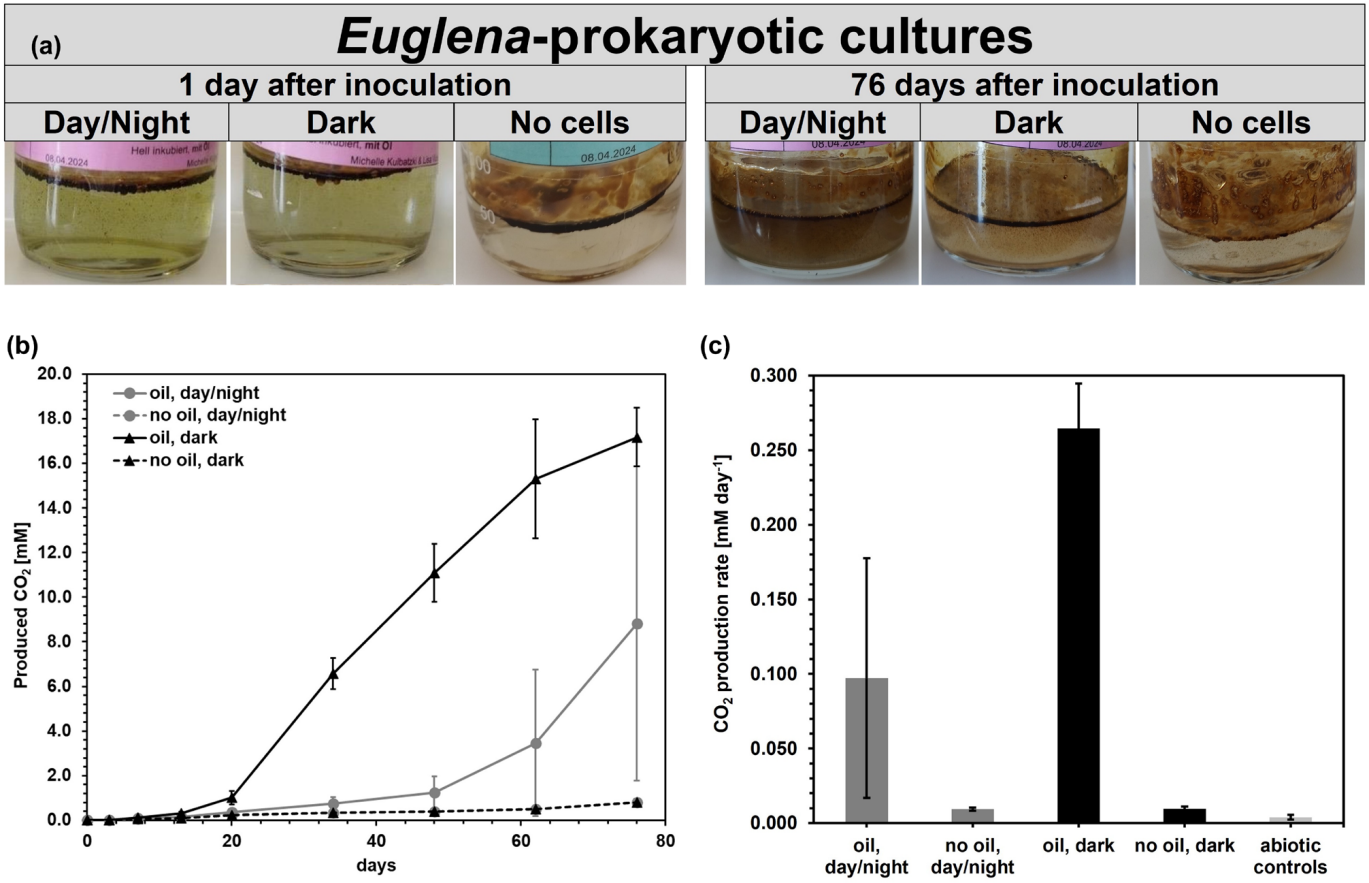
Experimental results from *Euglena*−prokaryotic oil degradation experiments. *Euglena*−prokaryotic cultures were incubated with day/night light rhythm and in the dark, each incubated with and without oil addition in order to test oil‐degradation potential. All conditions were tested in biological triplicates indicated by the error bars (standard deviation). (a) Photographs of chosen cultures and an abiotic control at day 1 and after 76 incubation days with produced oil snow incubated at different light regimes. (b) Mean produced CO_2_ (mM) over incubation time. Solid lines represent the *Euglena* culture incubations with oil addition and dotted lines without the addition of oil. (c) Mean CO_2_ production rates (mM · d^−1^). Error bars in b and c represent the standard deviation of three biological replicates.

The highest CO_2_ production of 17.2 ± 1.3 mM and a CO_2_ production rate of 0.26 ± 0.03 mM CO_2_ · d^−1^ (*R* ^2^ = 0.97) were observed for *Euglena* cultures incubated in the complete dark. *Euglena* cultures incubated with day/night light cycle produced approximately two times less CO_2_ (8.8 mM ± 7.04) at an approximately three times slower rate (0.097 ± 0.08 mM CO_2_ · d^−1^, *R* ^2^ = 0.73, Figure 1b,c, see raw data in Data S4), both being significantly different from each other (two‐tailed paired Student's *t*‐test: *p* = 0.008, Data S5). Based on the observed CO_2_ production, we estimated potential degradation rates under the tested conditions. Assuming constant degradation of the bioavailable fraction, a rough estimate suggests that the culture would require about 9 months to degrade 1 g of light oil under continuous darkness and about 2 years under day/night illumination (calculation according to Walter et al., 2026). However, these estimates should be interpreted with caution, as crude oil is a complex mixture of compounds with different biodegradability. The observed activity likely reflects the preferential transformation of more labile and bioavailable fractions (e.g., alkanes and low‐molecular‐weight polycyclic aromatic hydrocarbons, PAHs). As these compounds are depleted, the residual oil becomes successively enriched in more recalcitrant components such as high‐molecular‐weight PAHs, resins, and asphaltenes, which are degraded more slowly. Consequently, degradation rates are expected to decrease over time and cannot be assumed to remain constant across the entire oil mixture. Therefore, the calculated rates cannot be extrapolated to the degradation of the entire crude oil mixture, and these estimates are intended only as illustrative approximations and not as predictive values for whole‐oil degradation.

The observed differences in oil degradation rates between light and dark conditions reflect most likely the metabolic flexibility of mixotrophic *Euglena* and rather less the activity of its associated prokaryotes potentially involved in hydrocarbon degradation. *Euglena* preferentially shifted toward phototrophic metabolism when light was available, leading to lower oil degradation rates. If oil degradation was primarily conducted by the associated prokaryotes, the observed difference between light and dark conditions (two‐sided Welch's *t*‐test, *p* = 0.0205) would be unexpected, as prokaryotic hydrocarbon degradation is typically independent of light availability, relying mainly on chemoheterotrophic pathways (Das & Chandran, 2011). However, the differences observed between light and dark incubations could also arise from indirect effects mediated by the algal host. Light‐dependent shifts in *Euglena*'s metabolism, such as photosynthetic oxygen evolution, excretion of organic carbon compounds, or changes in redox and nutrient conditions, may substantially influence the activity and composition of associated prokaryotic communities (McGenity et al., 2012; Sun et al., 2022), which we did not see in our oil‐treated samples.

Prokaryotic communities, associated with *Euglena*, were characterized and compared via 16S rRNA gene amplicon sequencing at the beginning and after 62 days of the degradation experiment (Figure 2). *Euglena* had only low diverse prokaryotic communities in its culture consisting of only 15–78 amplicon sequence variants (ASVs; mean: 40 ± 1.5, Data S11) dominated mostly by the seven ASVs: ASV2_*Acidovorax* (blue), ASV10_*Nevskia* (light brown), ASV12_*Parablastomonas* (yellow), ASV7_*Herbaspirillum* (blue), ASV13_*Sediminibacterium* (light green), ASV6_*Caulobacter* (green), and ASV9_*Methylobacterium‐Methylorubrum* (purple). *Acidovorax* and *Nevskia* are known to be associated with oil‐contaminated environments and hydrocarbon/PAH degradation (Sjoholm et al., 2022; Yu et al., 2018); among the other ASVs, we could not find clear information. Incubations without the addition of oil were slightly more diverse after 62 days (observed ASVs: 46 ± 19, Shannon index: 1.58 ± 0.38) in comparison to oil‐treated cultures (observed ASVs: 30.4 ± 15, Shannon index: 1.35 ± 0.32), which could be an indicator for selectivity or toxicity of oil. Microbial communities showed partial overlap between day/night and dark oil incubations (Figure 2) but were significantly more similar within treatments than between, as indicated by lower within‐treatment Bray–Curtis dissimilarities (mean = 0.48) compared to between‐treatment dissimilarities (mean = 0.71; Wilcoxon test, *p* = 0.002). An overall permutational analysis of variance confirmed significant differences in community composition among treatments (*R* ^2^ = 0.57, *p* = 0.001, Data S13, Figure 2b), while homogeneity of dispersion was not violated (PERMDISP, *p* = 0.90). Despite this, pairwise differences were not statistically significant after multiple‐testing correction, and potential effects of *Euglena* on prokaryotic growth, and metabolism could therefore not be conclusively resolved within the experimental framework.

**FIGURE 2:**
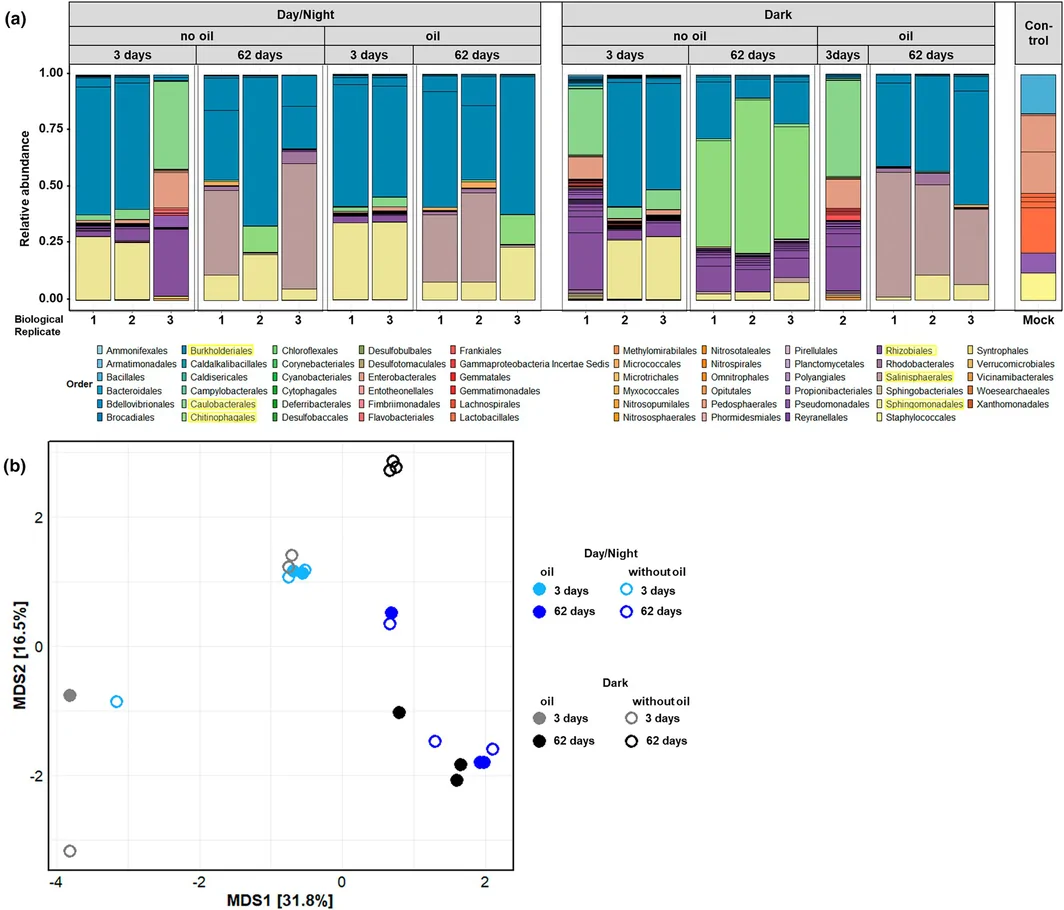
Microbial community compositions of *Euglena* sp. associated prokaryotes at beginning (3 days) and end (62 days) of the experiment incubated at day/night light cycle and solely in the dark each non‐treated or incubated with light oil as bar plot (a), in yellow highlighted the taxonomic orders with most abundant ASVs. (b) Principal component analysis of microbial community compositions of the different treatments. Each data point represents one culture microbial community. Light blue (3 days) and dark blue (62 days) indicate day/night‐incubated communities, while gray (3 days) and black (62 days) represent dark‐incubated communities. Filled circles correspond to oil‐treated cultures, whereas open circles indicate cultures without oil treatment.

The substantial reduction in degradation rate under day/night conditions, together with observed differences in microbial community compositions, suggests that light‐dependent processes influenced hydrocarbon degradation. This process might indicate a contribution of *Euglena*, potentially linked to its heterotrophic metabolism and interactions with heterotrophic partners; the specific role of *Euglena* cannot be conclusively resolved based on the presented data.

Pairwise comparisons between individual groups were not significant after Bonferroni correction, potentially reflecting the small sample sizes per group and the conservative nature of the multiple testing adjustment (Data S13).

The oil degradation observed in incubations containing *Euglena* sp. and its associated prokaryotic community was evident and quantifiable. These results highlight the potential of *Euglena* sp. culture (SLN082) in bioremediation applications and could become important for gaining knowledge for future bioremediation projects for sustainable and fast cleaning of human‐caused oil spills.

## AUTHOR CONTRIBUTIONS

**Michelle Kulbatzki:** Formal analysis (equal); investigation (equal); methodology (equal); writing – review and editing (supporting). **Hilal Kaya:** Formal analysis (supporting); investigation (supporting); methodology (supporting); writing – review and editing (supporting). **Sabina Marks:** Conceptualization (equal); data curation (equal); formal analysis (equal); investigation (equal); methodology (equal); project administration (equal); resources (equal); supervision (equal); validation (equal); writing – review and editing (equal). **Lisa Voskuhl:** Conceptualization (equal); data curation (equal); formal analysis (equal); funding acquisition (equal); investigation (equal); methodology (equal); project administration (equal); resources (equal); supervision (equal); validation (equal); visualization (equal); writing – original draft (lead); writing – review and editing (equal).

## Supporting information


**Data S1.** Supplementary data sets including sample information.
**Data S2.** Sampling days.
**Data S3.** pH measurements during the incubation experiments.
**Data S4.** Raw data and calculations of CO_2_ measurements for the RSIL.
**Data S5.** CO_2_ plots.
**Data S6.** An exemplary RSIL calculation.
**Data S7.** 16S rRNA gene amplicon sequencing data comprising metadata.
**Data S8.** Taxonomy table.
**Data S9.** Absolute ASV abundances.
**Data S10.** Relative ASV abundances.
**Data S11.** Alpha diversity metrics.
**Data S12.** Theoretical and measured mock community compositions used as positive controls.
**Data S13.** Calculated PERMANOVA dissimilarities.

**Figure S1.** Natural oil seep in a forest in Wietze, Lower Saxony. (A) Oil (black) seeps naturally out of the ground. (B) Spot where *Euglena* sp. (SLN082) culture was isolated from the soil 200 cm away from the main seep.
**Figure S2.** Microscopy picture of *Euglena* sp. culture (SLN082) made by Frank Fox.

## Data Availability

All relevant raw data are provided in the supplement data and raw sequencing data were deposited in the NCBI database under BioProject number PRJNA1367553. The *Euglena* sp. culture (SLN082) used in this study is available from the corresponding authors upon request.
