# Supplementary material for: Euglena sp. and its associated prokaryotic community degrade light crude oil
Source: J Phycol. 2026 Sep 8;62(5):1417–22. doi: 10.1111/jpy.70227 (PMC13659433; doi:10.1111/jpy.70227)
Supplement: Supplementary file 1 [file JPY-62-1417-s001.docx]

**Supplement**

***Euglena* sp. and its associated prokaryotic community degrade light crude oil**

Michelle Kulbatzki^1,2^, Hilal Kaya^1,2^, Sabina Marks^2,3*^, Lisa Voskuhl^1,3*^

^1^University of Duisburg-Essen, Faculty of Chemistry, Environmental Microbiology and Biotechnology (EMB), Microbiology of Ecotones

^2^University of Duisburg-Essen, Faculty of Biology, ZMB, Chemische Biologie

^3^Centre for Water and Environmental Research (ZWU), University of Duisburg-Essen, Essen, Germany

*Corresponding authors:

Sabina Marks sabina.marks@uni-due.de and Lisa Voskuhl lisa.voskuhl@uni-due.de

**Materials and methods**

**Environmental sampling and algae isolation**

Environmental sampling occurred on 31 August 2023 in a forest in Wietze (Lower Saxony, Germany, GPS: 52.666437,9.818928). *Euglena* sp. (SLN082) was isolated from a soil sample, collected 200 cm away from the natural oil seep (Figure S1, (Walter et al., 2025)).

Figure S1 Natural oil seep in a forest in Wietze, Lower Saxony. A) Oil (black) seeps naturally out of the ground. B) Spot where *Euglena* sp. (SLN082) culture was isolated from the soil 200 cm away from the main seep.

All following procedures were carried out under sterile conditions in a laminar flow cabinet. A spoon of the taken soil sample was put into a sterile petri dish filled with Waris-H growth medium (McFadden & Melkonian, 2019). The dish was incubated for a few days at a 14/10 hours day/night light cycle (< 50 ⋅ µmol photons ⋅ m^−2^⋅ s^−1^) at 21 °C. Individual algal cells were manually isolated under an inverted light microscope using sterile glass capillaries and transferred into fresh culture medium three-times for washing. The resulting single-cell–derived cultures were incubated under standard growth conditions and regularly monitored for growth. To maintain viability and obtain stable algal isolates with its associated microbiome (non-axenic), cultures were sub-cultured into fresh medium every 3 months. Selection of the algal strain used in this study was based on preliminary screenings. Several algal isolates were evaluated for their ability to grow in Waris-H medium supplemented with 10 mM bicarbonate (NaHCO_3_, needed for RSIL) and crude oil. Before addition of the algae the pH of the medium was adjusted with sterile 1 M hydrochloric acid to pH 7. *Euglena* sp. (SLN082) exhibited stable growth and a healthy morphology under these conditions and was selected for oil biodegradation experiments. The *Euglena* sp. culture (SLN082, Figure S2) used in this study is available from the corresponding authors upon request.

Figure S2 Microscopy picture of *Euglena* sp. culture (SLN082) made by Frank Fox.

Crude light oil

Light oil was provided by the oil and gas company Aker BP ASA and was obtained in 2016 from a Norwegian offshore reservoir at a depth of 3700 m. It has a density of 847.6 kg ⋅ m⁻³ at 15 °C and an API gravity of 37° (Beilig et al., 2024; Walter et al., 2026). The light oil was stored in the dark at room temperature until use. The light crude oil was autoclaved for 20 min at 121 °C and left to sit for 1 week. It was then autoclaved a second time to ensure the inactivation of any potential spore forming organisms.

Oil degradation experiment set up and design

Before the start of the experiment a total volume of 750 mL *Euglena* sp. (SLN082) was pre-cultivated. For the degradation experiment, 750 mL of fresh Waris-H with bicarbonate medium (10 mM, non-^13^C-labeled) was mixed with the 750 mL *Euglena* pre-culture. Twelve sterile 250-mL glass bottles (Schott) were filled with 100 mL of the prepared *Euglena* sp. culture and following cultures conditions were set up in biological triplicates. Six glass bottles were amended with 1 mL of sterilized light crude oil. Three bottles were incubated under a day/night light cycle, and three were maintained in the dark. An additional six bottles served as no-oil controls, with three incubated under the same day/night cycle and three kept in the dark. Four abiotic medium controls (no algae; containing Waris-H medium and 10 mM bicarbonate; final volume 100 mL) were also prepared. Two of these controls were amended with 1 mL of light crude oil, and two received no oil. For both the oil-amended and no-oil abiotic controls, one bottle was incubated under a day/night light cycle, and the other was kept in the dark.

All bottles were sealed gastight with sterilized butyl stoppers and screw caps to secure the stoppers and permit sampling through them. After the bottles were closed, 1 mL of sterilized ^13^C-labeled bicarbonate buffer to a final concentration of 10 mM and 14 atom % of ^13^C was added with a sterile syringe and needle, which had been flushed with CO_2_-free synthetic air (ALPHAGAZ™ 2 air, 20.9 % O₂ in N₂, Air Liquide, Paris, France). Dark incubated bottles were wrapped into aluminum foil and incubated in the dark. The light incubations were placed on an LED light panel with day (14h) /night (10h) light cycle, the LED panel had a light intensity of 0.187 mW/cm^2^ measured via Spectri 80 spectroradiometer (SSL Resource, Finland). The dark incubations were included to distinguish the contribution of phototrophic and heterotrophic metabolism of the mixotrophic *Euglena* sp. to CO_2_ production during potential oil degradation.

Sampling of microcosms

All bottles were sampled under sterile conditions. Sampling was performed close to a Bunsen burner flame, and ethanol was applied to the butyl stoppers and ignited before piercing the stopper. To avoid introduction of CO_2_ into the bottles a sterile Hungate needle was flushed three times with synthetic air before being inserted through the butyl stoppers. From each culture bottle, in total 6 mL of the aqueous phase was sampled with two syringes. The 3 mL of the first syringe was filtered, in order to prevent oil from entering the CO_2_ vials (0.2 μm, Filtropur S0.2, dead volume ~0.7 mL, Sarstedt AG & Co. KG, Nümbrecht, Germany), ~1.5 mL of the sample was used to account for the dead volume of the filter, and per sample 500 μL were injected into each of three prepared 12 mL test tubes for technical replicates (Labco United, United Kingdom) for the CO_2_-measurement. For preparation, the 12-mL test tubes were pre-filled with 50 μL of 85 % phosphoric acid, sealed with a screw cap containing a butyl septum, and flushed with CO_2_-free synthetic air (6.0 purity, Air Liquide, Germany) before the sampling. The second syringe with the remaining 3 mL of the aqueous phase was left unfiltered and distributed into three reaction tubes: 1 mL for pH measurement (Mettler Toledo FiveEasy F20, Mettler Toledo GmbH, Switzerland, equipped with electrode: Mettler Toledo Type No. 51343164, Ser No. L23/136) and 2 mL into a DNase-free reaction tube (Sarstedt AG & Co. KG, Nümbrecht, Germany) for 16S rRNA gene amplicon sequencing. Samples for 16S sequencing were stored at – 70 °C afterwards. Sampling was conducted at days 0, 3, 7, 13, 20, 34, 48, 62, and 76 days of incubation (Data S2).

Reverse stable isotope labeling (RSIL)

Oil degradation and degradation rates were determined using the RSIL method. This method has been shown to be sensitive for measuring oil degradation and has been reported in several previous studies (Beilig et al., 2024; Beilig et al., 2025; Dong et al., 2019; Pannekens et al., 2021 ). Here we used the G2131-i Isotope and Gas concentration analyzer (Picarro, USA) which measures the change in δ^13^C/^12^C isotopic ratio of the CO_2_ evolved from a substrate during microbial degradation. An advantage of this method is that is does not require knowledge of the substrate’s chemical composition (here light oil), because only the change in the δ^13^C/^12^C isotopic ratio from the substrate released CO_2_ is measured (Beilig et al., 2024; Pannekens et al., 2021; Walter et al., 2025). In other words, the reported CO₂ production rates represent the total amount of CO₂ derived from substrate degradation over time. Euglena photosynthesis is not influencing the RSIL results, as during photosynthesis it removes CO_2_ without the change of the δ^13^C/^12^C isotopic composition of the CO_2_ which was produced from substrate degradation. The preferred consumption of ^12^C (fractionation) can be neglected in comparison to the δ^13^C/^12^C change due to ^12^C released by substrate mineralization. Literature reports that Euglena fractionates δ¹³C by about –20 ± 5 ‰ under photosynthetic conditions (Taipale et al., 2015), whereas our observed δ¹³C/¹²C shifts between time points were much larger, ranging from -17,925.5 to -2.616 ‰ (n = 269; median = -330.41 ‰; mean = -1028.97 ± 2103.89 ‰) along 76 days of incubation. These values are several orders of magnitude greater than the isotopic fractionation typically associated with biological carbon fixation. Additionally, fractionation would lead to an underestimation of produced CO_2_ in the RSIL experiments. The detailed calculation is explained in Data S6.

*Euglena*: DNA extraction and 18S and 16S rRNA gene sanger sequencing

18S rRNA gene sequencing of *Euglena* sp. was performed to confirm the taxonomic identity of the isolated culture at the molecular level. DNA was extracted from 100 mL of algal culture using the my-Budget DNA Mini Kit (Bio-Budget Technologies GmbH). Cultures were centrifuged in 50 mL tubes at 5,000 rpm for 3 min, and the resulting pellets were transferred to new tubes and centrifuged at 11,000 × *g* until minimal supernatant remained. The sample was then transferred to the pre-cooled mortar and ground thoroughly with liquid nitrogen until a fine, clump-free powder was obtained and followed the protocol.

DNA from algal cultures was used to amplify the 18S rRNA gene via PCR with forward primer 5′-AAT CTG GTT GAT CCT GCC AG-3′ and reverse primer 5′-TGA TCC TTC TGC AGG TTC ACC TAC-3′ (Preisfeld et al., 2000), yielding a length of 1.7-1.8 kbp. PCR reactions contained 10x buffer (2.5 µL), 2.5 mM dNTPs (0.75 µL), 10 pmol ⋅ µL^−1^ primers (each 1.5 µL), DreamTaq polymerase (0.2 µL), and 1 µL DNA template and 17.5 µL PCR water. Thermal cycling consisted of initial denaturation at 95 °C for 3 min, followed by 30 cycles of denaturation (95 °C, 30 s), annealing (57 °C, 30 s), and extension (72 °C, 2 min), with a final extension at 72 °C for 7 min. The PCR products were sequenced externally by Microsynth AG (Balgach, Schweiz). Raw sequencing data are available under NCBI BioProject PRJNA1367553.

Associated microbiome: 16S rRNA gene amplicon library preparation

DNA was extracted from each incubation at the beginning of the experiment after 3 days and after 62 days of inoculation using the DNeasy PowerLyzer PowerSoil Kit (Qiagen, Germany) following the manufacturer’s instructions with minor modifications as reported before (Beilig et al., 2025; Walter et al., 2025). The V4 region of the bacterial and archaeal 16S rRNA genes was amplified using the primer set S-D-Arch-0519-a-S-15 (5′-CAGCCGCCGCGGTAA-3′) and S-D-Bact-0785-b-A-18 (5′-GACTACHVGGGTATCTAATCC-3′; Takahashi et al., 2014) at a final concentration of 0.2 µM each, following a touchdown PCR protocol. Thermocycling was initiated with 5 min at 95 °C, followed by 10 touchdown cycles consisting of 30 s at 95 °C, 45 s at 60–55 °C (decreasing by 0.5 °C per cycle), and 60 s at 72 °C. This was followed by 30 cycles of 30 s at 95 °C, 45 s at 55 °C, and 60 s at 72 °C, with a final extension step of 5 min at 72 °C. Amplicon PCR products were verified by agarose gel electrophoresis using 1 % (w/v) gels. Amplicons were purified following the Illumina protocol, employing 16 μL of MagSi-NGS Prep-Plus magnetic beads per 20 μL of PCR product (Steinbrenner Laborsysteme GmbH, Mannheim, Germany). The purified products were subsequently used as templates for index PCR using the Nextera XT Index Kit v2 Set D (FC-131-2004; Illumina, United States). Index PCR was performed with an initial denaturation at 95 °C for 3 min, followed by 10 cycles of 95 °C for 30 s, 55 °C for 45 s, and 72 °C for 60 s, and a final extension at 72 °C for 5 min. The resulting index PCR products were again checked by agarose gel electrophoresis and purified using magnetic beads as described above. DNA concentrations were determined using the Qubit dsDNA High Sensitivity assay kit (Invitrogen, United States), and samples were normalized to 4 ng ⋅ μL⁻¹ with 10 mM Tris-Cl (pH 8.5; buffer EB, Qiagen, Germany). Final DNA concentrations were normalized to 10 ng ⋅ µL⁻¹ and pooled into a single sequencing library. Negative DNA-extraction and PCR controls, as well as a commercial microbial community positive control (ZymoBIOMICS® D6300), were included, for results see Data S12. Sequencing was performed on an Illumina MiSeq platform (V2 500-cycle) by CeGaT GmbH (Tübingen, Germany), including demultiplexing and adapter trimming. Sequence data was processed in R using DADA2 v1.30.0 and phyloseq v1.52.0. Forward and reverse reads were trimmed to 240 and 160 bp, respectively, with filterAndTrim(fnFs, filtFs, fnRs, filtRs, truncLen=c(240,160), trimLeft=c(14,21), maxN=0, maxEE=c(2,2), truncQ=2, rm.phix=TRUE, compress=TRUE, multithread=FALSE). Reads were merged, chimeras removed, and taxonomic assignment performed using the SILVA SSU138.2 database. Amplicon Sequence Variants (ASVs) with fewer than 10 reads or classified as “NA”; chloroplast, or mitochondrial sequences were excluded. To ensure comparability across samples, all datasets were rarefied to 4304 reads. Three samples (t0_E2.1, t0_E2.3, and NegCo) did not meet this threshold after quality filtering and were therefore excluded from downstream analyses. This resulted in reduced replicate numbers for the affected conditions (Data S7). Within- and between-group dissimilarities were calculated from a Bray–Curtis distance matrix. Pairwise distances were extracted and classified as within-treatment or between-treatment comparisons based on group membership. Mean and standard deviation of each category were computed. Statistical differences between within- and between-treatment distances were tested using a Wilcoxon rank-sum test. Community dispersion among groups was assessed using the betadisper function, and differences in multivariate dispersion were evaluated using a one-way ANOVA on the resulting distances (see Data S13).

Raw sequencing data are available under NCBI BioProject PRJNA1367553.

**References**

Beilig, S., Pannekens, M., Voskuhl, L., & Meckenstock, R. U. (2024). Assessing anaerobic microbial degradation rates of crude light oil with reverse stable isotope labelling and community analysis. *Front Microbiomes*, *3*. <https://doi.org/10.3389/frmbi.2024.1324967>

Beilig, S., Voskuhl, L., Geydirici, I., Tintrop, L. K., Schmidt, T. C., & Meckenstock, R. U. (2025). Coexistence of methanogenesis and sulfate reduction in a sulfate-adapted enrichment culture from an oil reservoir. *Applied and Environmental Microbiology*, *91*(12), e0014125. <https://doi.org/10.1128/aem.00141-25>

Dong, X., Bäcker, L. E., Rahmatullah, M., Schunk, D., Lens, G., & Meckenstock, R. U. (2019). Quantification of microbial degradation activities in biological activated carbon filters by reverse stable isotope labelling. *AMB express*, *9*(1), 109. <https://doi.org/10.1186/s13568-019-0827-0>

McFadden, G. I., & Melkonian, M. (2019). Use of Hepes buffer for microalgal culture media and fixation for electron microscopy. *Phycologia*, *25*(4), 551–557. <https://doi.org/10.2216/i0031-8884-25-4-551.1>

Pannekens, M., Voskuhl, L., Mohammadian, S., Köster, D., Meier, A., Köhne, J. M., Kulbatzki, M., Akbari, A., Haque, S., & Meckenstock, R. U. (2021). Microbial degradation rates of natural bitumen. *Environmental Science & Technology*, *55*(13), 8700–8708. <https://doi.org/10.1021/acs.est.1c00596>

Preisfeld, A., Berger, S., Busse, I., Liller, S., & Ruppel, H. G. (2000). Phylogenetic analyses of various euglenoid taxa (euglenozoa) based on 18s rDNA sequence data. *Journal of Phycology*, *36*(1), 220–226. <https://doi.org/10.1046/j.1529-8817.2000.99091.x>

Taipale, S. J., Peltomaa, E., Hiltunen, M., Jones, R. I., Hahn, M. W., Biasi, C., & Brett, M. T. (2015). Inferring phytoplankton, terrestrial plant and bacteria bulk δ¹³C values from compound specific analyses of lipids and fatty acids. *PLoS ONE*, *10*(7), e0133974. <https://doi.org/10.1371/journal.pone.0133974>

Takahashi, S., Tomita, J., Nishioka, K., Hisada, T., & Nishijima, M. (2014). Development of a prokaryotic universal primer for simultaneous analysis of Bacteria and Archaea using next-generation sequencing. *PLoS ONE*, *9*(8), e105592. <https://doi.org/10.1371/journal.pone.0105592>

Walter, R., Willemsen, L., & Voskuhl, L. (2025). A microbial seedbank from a natural oil seep accelerates hydrocarbon degradation in freshwater oil spills. *submitted to Environ Microbiome*.

Walter, R., Willemsen, L., & Voskuhl, L. (2026). *A microbial seedbank from a natural oil seep accelerates hydrocarbon degradation in freshwater oil spills*. BioRxiv. <https://doi.org/10.64898/2026.03.16.712059>
